# (1R*,2R*,5R*,6S*)-6-Bromo-9-oxabi­cyclo­[3.3.1]nonan-2-ol

**DOI:** 10.1107/S2414314625008545

**Published:** 2025-10-09

**Authors:** Lea Simon, Heiner Detert, Dieter Schollmeyer

**Affiliations:** a University Mainz, Duesbergweg 10-14, 55099 Mainz, Germany; Goethe-Universität Frankfurt, Germany

**Keywords:** crystal structure, heterocycle, bromine, hydrogen bridge

## Abstract

Both six-membered rings in the title bi­cyclo­[3.3.1] system, C_8_H_13_BrO_2_, adopt a chair conformation. Hydrogen bonds from the hy­droxy group to the ether bridge connect the mol­ecules into zigzag chains: single-enantiomer chains propagating along the *b*-axis direction form the crystal.

## Abstract 2

Both six-membered rings in the title bi­cyclo­[3.3.1] system, adopt a chair conformation. Hydrogen bonds from the hy­droxy group to the ether bridge connect the mol­ecules into zigzag chains: single-enantiomer chains propagating along the *b*-axis direction form the crystal.

## Graphical abstract

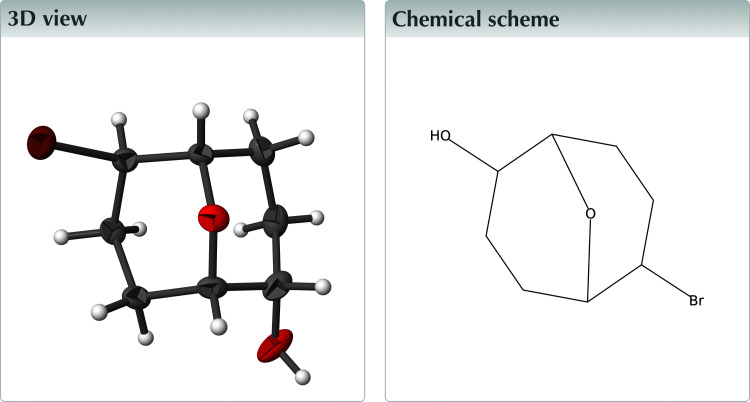

## Structure description

**Figure 1:**
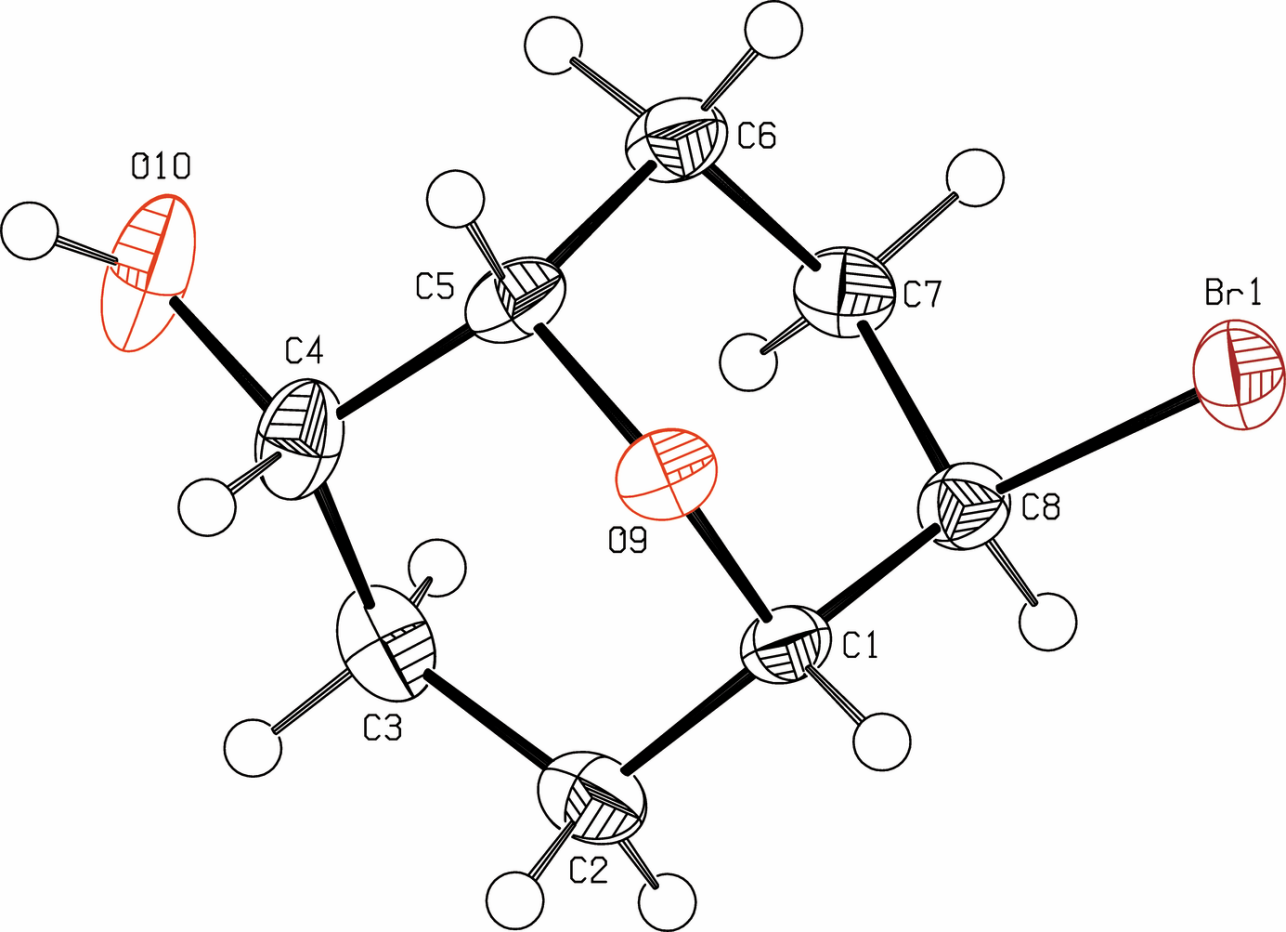
View (Spek, 2009 ▸) of the title compound. Displacement ellipsoids are drawn at the 50% probability level.

**Figure 2:**
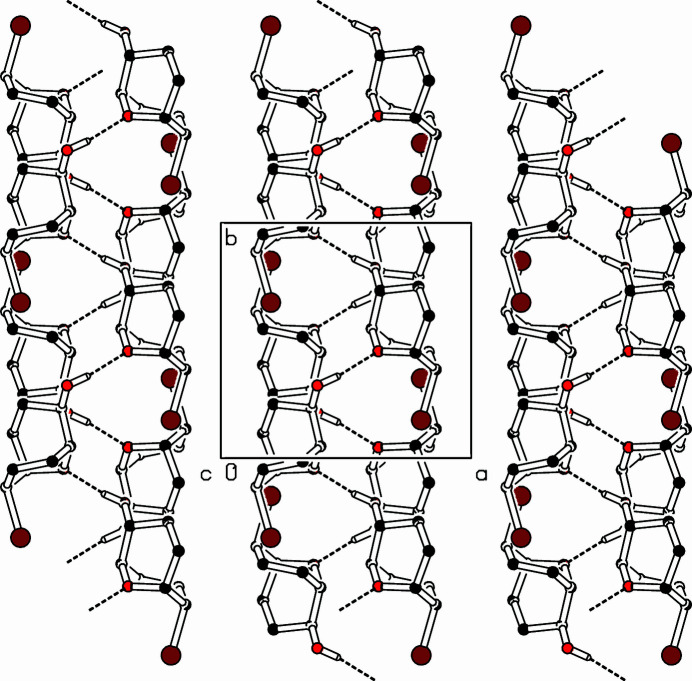
Part of the packing diagram. View along the *c*-axis direction (Spek, 2009 ▸). Hydrogen atoms bonded to C atoms are omitted for clarity.

The title compound, C_8_H_13_BrO_2_ (Fig. 1 ▸), was prepared as part of a project on medium-sized rings (Detert *et al.*, 1994 ▸; Detert & Meier 1997*a* ▸,*b* ▸) and transannular reactions (Detert *et al.*, 1992 ▸, Kraemer *et al.*, 2009 ▸; Meier *et al.*, 2009 ▸). The oxabi­cyclo­[3.3.1] framework is close to being perfectly *C*_2*v*_ symmetrical with both six-membered rings in a chair conformation. The centrosymmetrical crystal is composed of two counter-directional chains generated by a twofold screw axis. Both of these zigzag chains run along the *b*-axis direction (Fig. 2 ▸). Each chain is composed of a single enanti­omer, the mol­ecules are connected *via* hydrogen-bond bridges (O10—H10⋯O9) with an O⋯O distance of 1.91 (6) Å and an O—H⋯O 164 (3)° angle. The chains are connected by C—H⋯O contacts (Table 1 ▸).

## Synthesis and crystallization

The synthesis of the title compound was performed by di­hydroxy­lation of 1,5-cyclo­octa­diene (Yates *et al.*, 1972 ▸), acetalization, addition of bromine (Schollmeyer *et al.*, 2020 ▸) and hydrolysis of the acetal concomitant with an intra­molecular nucleophilic substitution of one bromine atom by a hydroxyl group according to Takahashi *et al.* (2000 ▸). (1R*,4S*,5S*,8R*)-4,5-Di­bromo-10,10-dimethyl-9,11-dioxabi­cyclo­[6.3.0]undecane was the main isomer (*ca* 10/1) of the bromination step. 2.50 g of the crude product were purified *via* silica column chromatography using a cyclohexane–ethyl acetate (1:10) and 2% triethylamine eluent. (1R*,4S*,5S*,8R*)-4,5-Dibromo-10,10-dimethyl-9,11-dioxabicyclo[6.3.0]undecane (4b) was obtained as a colorless oil (1.03 g, 3.01 mmol, 46% of theory). Then 0.90 g (2.63 mmol) of this mixture were dissolved in THF (4 ml), hydro­chloric acid (1*M*, 4 ml) was added and the mixture was stirred at 323 K for 16 h while the reaction progress was monitored *via* TLC. After full conversion, the mixture was neutralized with saturated aqueous NaHCO_3_ and extracted with ethyl acetate (4 × 25 ml). The combined organic layers were washed with brine (2 × 30 ml), dried over Na_2_SO_4_, and concentrated *in vacuo*. The residue was purified by column chromatography using cyclo­hexane-ethyl acetate as an eluent (1:1, *R*_f_ = 0.32). (1R*,2R*,5R*,6S*)-6-Bromo-9-oxabi­cyclo­[3.3.1]nonan-2-ol was obtained as a crystalline, colorless solid (0.54 g, 2.44 mmol, 93% of theory) with m.p. = 341–344 K. Spectroscopic data: (assignment of signals follows IUPAC nomenclature): IR (ATR): ν (cm^−1^) = 3390*mb*, 2940*s*,1733*w*, 1481*m*, 1443*m*, 1232*m*, 1084*m*, 1028*s*, 978*m*, 895*s*, 871*s*, 849*s*. ^1^H-NMR (300 MHz, CDCl_3_): δ = 4.37–4.28 (*m*, 1H, 1-H), 4.14–3.95 (*m*, 2H, 2-H, 6-H), 3.90 (*t*, *J* = 5.92 Hz, 1H, 5-H), 2.56 (*s*, 1H, OH), 2.50–2.15 (*m*, 2H, 7-H, 8-H), 2.19–1.92 (*m*, 3H, 3-H, 4-H, 8′-H), 1.92–1.64 (*m*, 3H, 3-H, 4-H, 7′-H). ^13^C-NMR (75 MHz, CDCl_3_) δ = 71.92 (2-C), 70.05 (5-C), 68.07 (6-C), 53.08 (1-C), 29.07 (8-C), 27.48 (4-C), 27.38 (3-C), 17.99 (7-C). LC–MS: *m*/*z* 221.000 [*M* + H]^+^; (calculated for C_8_H_14_O_2_Br [*M* + H]^+^: 221.018).

## Refinement

Crystal data, data collection and structure refinement details are summarized in Table 2 ▸.

## Supplementary Material

Crystal structure: contains datablock(s) I, global. DOI: 10.1107/S2414314625008545/bt4181sup1.cif

Structure factors: contains datablock(s) I. DOI: 10.1107/S2414314625008545/bt4181Isup2.hkl

Supporting information file. DOI: 10.1107/S2414314625008545/bt4181Isup3.cml

CCDC reference: 2492093

Additional supporting information:  crystallographic information; 3D view; checkCIF report

## Figures and Tables

**Table 1:** Hydrogen-bond geometry (Å, °)

*D*—H⋯*A*	*D*—H	H⋯*A*	*D*⋯*A*	*D*—H⋯*A*
C2—H2*B*⋯O10^i^	0.95 (4)	2.45 (4)	3.378 (5)	164 (3)
O10—H10⋯O9^ii^	0.83 (6)	1.91 (6)	2.732 (4)	166 (5)

Symmetry codes: (i) 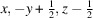; (ii) 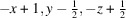.

**Table 2:** Experimental details

Crystal data	
Chemical formula	C_8_H_13_BrO_2_
*M*_r_	221.09
Crystal system, space group	Monoclinic, *P*2_1_/*c*
Temperature (K)	120
*a*, *b*, *c* (Å)	9.5387 (7), 8.8911 (8), 10.2453 (7)
β (°)	97.088 (6)
*V* (Å^3^)	862.26 (12)
*Z*	4
Radiation type	Mo *K*α
μ (mm^−1^)	4.72
Crystal size (mm)	0.60 × 0.35 × 0.14

Data collection	
Diffractometer	Stoe *IPDS* 2T
Absorption correction	Integration (*X-RED32*; Stoe & Cie, 2020 ▸)
*T*_min_, *T*_max_	0.196, 0.519
No. of measured, independent and observed [*I* > 2σ(*I*)] reflections	4579, 2057, 1808
*R*_int_	0.023
(sin θ/λ)_max_ (Å^−1^)	0.659

Refinement	
*R*[*F*^2^ > 2σ(*F*^2^)], *wR*(*F*^2^), *S*	0.042, 0.089, 1.27
No. of reflections	2057
No. of parameters	146
H-atom treatment	Only H-atom coordinates refined
Δρ_max_, Δρ_min_ (e Å^−3^)	0.79, −0.54

Computer programs: *X-AREAWinXpose*, *Recipe* and *Integrate* (Stoe & Cie, 2020 ▸), *SHELXT2014* (Sheldrick, 2015*a* ▸), *SHELXL2019/2* (Sheldrick, 2015*b* ▸) and *PLATON * (Spek, 2009 ▸).

## full crystallographic data

### (1R*,2R*,5R*,6S*)-6-Bromo-9-oxabicyclo[3.3.1]nonan-2-ol Crystal data

**Table:**

C_8_H_13_BrO_2_	*F*(000) = 448
*M**_r_* = 221.09	*D*_x_ = 1.703 Mg m^−^^3^
Monoclinic, *P*2_1_/*c*	Mo *K*α radiation, λ = 0.71073 Å
*a* = 9.5387 (7) Å	Cell parameters from 8646 reflections
*b* = 8.8911 (8) Å	θ = 2.8–28.4°
*c* = 10.2453 (7) Å	µ = 4.72 mm^−^^1^
β = 97.088 (6)°	*T* = 120 K
*V* = 862.26 (12) Å^3^	Block, colorless
*Z* = 4	0.60 × 0.35 × 0.14 mm

### (1R*,2R*,5R*,6S*)-6-Bromo-9-oxabicyclo[3.3.1]nonan-2-ol Data collection

**Table:**

Stoe IPDS 2T diffractometer	2057 independent reflections
Radiation source: sealed X-ray tube, 12 x 0.4 mm long-fine focus	1808 reflections with *I* > 2σ(*I*)
Detector resolution: 6.67 pixels mm^-1^	*R*_int_ = 0.023
rotation method, ω scans	θ_max_ = 27.9°, θ_min_ = 3.0°
Absorption correction: integration (X-Red32; Stoe & Cie, 2020)	*h* = −11→12
*T*_min_ = 0.196, *T*_max_ = 0.519	*k* = −11→10
4579 measured reflections	*l* = −13→13

### (1R*,2R*,5R*,6S*)-6-Bromo-9-oxabicyclo[3.3.1]nonan-2-ol Refinement

**Table:**

Refinement on *F*^2^	Primary atom site location: dual
Least-squares matrix: full	Hydrogen site location: difference Fourier map
*R*[*F*^2^ > 2σ(*F*^2^)] = 0.042	Only H-atom coordinates refined
*wR*(*F*^2^) = 0.089	*w* = 1/[σ^2^(*F*_o_^2^) + (0.0222*P*)^2^ + 1.9676*P*] where *P* = (*F*_o_^2^ + 2*F*_c_^2^)/3
*S* = 1.27	(Δ/σ)_max_ < 0.001
2057 reflections	Δρ_max_ = 0.79 e Å^−^^3^
146 parameters	Δρ_min_ = −0.54 e Å^−^^3^
0 restraints	

### (1R*,2R*,5R*,6S*)-6-Bromo-9-oxabicyclo[3.3.1]nonan-2-ol Special details

**Table:**

Geometry. All esds (except the esd in the dihedral angle between two l.s. planes) are estimated using the full covariance matrix. The cell esds are taken into account individually in the estimation of esds in distances, angles and torsion angles; correlations between esds in cell parameters are only used when they are defined by crystal symmetry. An approximate (isotropic) treatment of cell esds is used for estimating esds involving l.s. planes.
Refinement. Hydrogen atoms were freely refined, except H1 and H5 which were refined with *U*(H)=1.2*U*_eq_(C). The H atoms of each CH_2_ group were given a common displacement parameter.

### (1R*,2R*,5R*,6S*)-6-Bromo-9-oxabicyclo[3.3.1]nonan-2-ol Fractional atomic coordinates and isotropic or equivalent isotropic displacement parameters (Å^2^)

**Table:**

	*x*	*y*	*z*	*U*_iso_*/*U*_eq_	
Br1	0.19929 (4)	0.83866 (4)	0.25179 (4)	0.03210 (13)	
C1	0.2223 (4)	0.5517 (4)	0.1306 (3)	0.0235 (7)	
H1	0.220 (4)	0.620 (4)	0.059 (4)	0.028*	
C2	0.1690 (4)	0.3953 (5)	0.0871 (4)	0.0306 (8)	
H2A	0.065 (4)	0.393 (5)	0.065 (4)	0.029 (7)*	
H2B	0.213 (4)	0.371 (5)	0.011 (4)	0.029 (7)*	
C3	0.2108 (4)	0.2714 (4)	0.1884 (4)	0.0334 (8)	
H3A	0.149 (5)	0.269 (5)	0.260 (4)	0.041 (9)*	
H3B	0.196 (5)	0.176 (5)	0.148 (5)	0.041 (9)*	
C4	0.3622 (4)	0.2883 (4)	0.2501 (4)	0.0316 (8)	
H4	0.431 (4)	0.266 (5)	0.185 (4)	0.032 (11)*	
C5	0.3972 (4)	0.4502 (4)	0.2913 (3)	0.0255 (7)	
H5	0.492 (4)	0.458 (5)	0.314 (4)	0.031*	
C6	0.3264 (4)	0.5148 (5)	0.4058 (3)	0.0277 (7)	
H6A	0.343 (5)	0.436 (5)	0.472 (5)	0.045 (9)*	
H6B	0.382 (5)	0.608 (6)	0.434 (4)	0.045 (9)*	
C7	0.1695 (4)	0.5486 (4)	0.3687 (3)	0.0262 (7)	
H7A	0.113 (4)	0.454 (5)	0.366 (4)	0.032 (8)*	
H7B	0.138 (4)	0.616 (5)	0.439 (4)	0.032 (8)*	
C8	0.1421 (4)	0.6236 (4)	0.2342 (3)	0.0247 (7)	
H8	0.044 (4)	0.631 (4)	0.201 (4)	0.020 (9)*	
O9	0.3704 (2)	0.5456 (3)	0.1771 (2)	0.0250 (5)	
O10	0.3857 (4)	0.1913 (4)	0.3610 (3)	0.0464 (8)	
H10	0.466 (6)	0.153 (6)	0.362 (5)	0.057 (16)*	

### (1R*,2R*,5R*,6S*)-6-Bromo-9-oxabicyclo[3.3.1]nonan-2-ol Atomic displacement parameters (Å^2^)

**Table:**

	*U*^11^	*U*^22^	*U*^33^	*U*^12^	*U*^13^	*U*^23^
Br1	0.0394 (2)	0.02595 (18)	0.03210 (19)	0.00348 (17)	0.00881 (14)	0.00081 (15)
C1	0.0223 (16)	0.0300 (18)	0.0177 (15)	0.0044 (14)	0.0005 (12)	0.0003 (13)
C2	0.0251 (18)	0.038 (2)	0.0291 (18)	−0.0019 (16)	0.0043 (15)	−0.0090 (15)
C3	0.033 (2)	0.0276 (18)	0.041 (2)	−0.0023 (16)	0.0112 (17)	−0.0056 (16)
C4	0.031 (2)	0.0299 (18)	0.0356 (19)	0.0081 (15)	0.0133 (16)	0.0093 (15)
C5	0.0185 (16)	0.0355 (19)	0.0219 (16)	0.0024 (14)	0.0006 (13)	0.0047 (14)
C6	0.0258 (18)	0.037 (2)	0.0197 (15)	0.0042 (16)	0.0017 (14)	0.0017 (14)
C7	0.0272 (18)	0.0313 (18)	0.0210 (16)	−0.0015 (15)	0.0072 (14)	0.0000 (13)
C8	0.0210 (16)	0.0287 (18)	0.0241 (16)	0.0013 (14)	0.0016 (13)	−0.0004 (13)
O9	0.0230 (12)	0.0299 (13)	0.0228 (11)	−0.0029 (10)	0.0051 (9)	0.0041 (10)
O10	0.0464 (19)	0.0454 (18)	0.0523 (18)	0.0253 (15)	0.0260 (15)	0.0253 (14)

### (1R*,2R*,5R*,6S*)-6-Bromo-9-oxabicyclo[3.3.1]nonan-2-ol Geometric parameters (Å, º)

**Table:**

Br1—C8	1.991 (4)	C4—H4	1.01 (4)
C1—O9	1.435 (4)	C5—O9	1.443 (4)
C1—C8	1.523 (5)	C5—C6	1.535 (5)
C1—C2	1.529 (5)	C5—H5	0.91 (4)
C1—H1	0.95 (4)	C6—C7	1.528 (5)
C2—C3	1.533 (6)	C6—H6A	0.97 (5)
C2—H2A	0.99 (4)	C6—H6B	1.01 (5)
C2—H2B	0.95 (4)	C7—C8	1.524 (5)
C3—C4	1.511 (6)	C7—H7A	1.00 (4)
C3—H3A	1.00 (5)	C7—H7B	1.01 (4)
C3—H3B	0.95 (5)	C8—H8	0.96 (4)
C4—O10	1.422 (4)	O10—H10	0.83 (6)
C4—C5	1.526 (6)		
			
O9—C1—C8	110.1 (3)	O9—C5—C6	110.5 (3)
O9—C1—C2	109.9 (3)	C4—C5—C6	117.6 (3)
C8—C1—C2	114.1 (3)	O9—C5—H5	104 (3)
O9—C1—H1	102 (3)	C4—C5—H5	108 (3)
C8—C1—H1	108 (2)	C6—C5—H5	107 (3)
C2—C1—H1	112 (2)	C7—C6—C5	113.1 (3)
C1—C2—C3	114.0 (3)	C7—C6—H6A	113 (3)
C1—C2—H2A	112 (2)	C5—C6—H6A	102 (3)
C3—C2—H2A	108 (2)	C7—C6—H6B	112 (3)
C1—C2—H2B	106 (2)	C5—C6—H6B	105 (3)
C3—C2—H2B	106 (2)	H6A—C6—H6B	111 (4)
H2A—C2—H2B	110 (3)	C8—C7—C6	111.4 (3)
C4—C3—C2	111.6 (3)	C8—C7—H7A	108 (2)
C4—C3—H3A	109 (3)	C6—C7—H7A	111 (2)
C2—C3—H3A	112 (3)	C8—C7—H7B	111 (2)
C4—C3—H3B	111 (3)	C6—C7—H7B	108 (2)
C2—C3—H3B	110 (3)	H7A—C7—H7B	108 (3)
H3A—C3—H3B	104 (4)	C1—C8—C7	113.6 (3)
O10—C4—C3	108.8 (3)	C1—C8—Br1	108.1 (2)
O10—C4—C5	110.1 (3)	C7—C8—Br1	108.9 (2)
C3—C4—C5	112.0 (3)	C1—C8—H8	110 (2)
O10—C4—H4	111 (2)	C7—C8—H8	114 (2)
C3—C4—H4	112 (2)	Br1—C8—H8	102 (2)
C5—C4—H4	104 (2)	C1—O9—C5	111.1 (2)
O9—C5—C4	108.6 (3)	C4—O10—H10	108 (4)
			
O9—C1—C2—C3	49.8 (4)	C5—C6—C7—C8	−42.9 (4)
C8—C1—C2—C3	−74.3 (4)	O9—C1—C8—C7	−53.9 (4)
C1—C2—C3—C4	−42.3 (4)	C2—C1—C8—C7	70.1 (4)
C2—C3—C4—O10	168.0 (3)	O9—C1—C8—Br1	67.1 (3)
C2—C3—C4—C5	46.0 (4)	C2—C1—C8—Br1	−168.9 (2)
O10—C4—C5—O9	−179.2 (3)	C6—C7—C8—C1	43.8 (4)
C3—C4—C5—O9	−58.1 (4)	C6—C7—C8—Br1	−76.8 (3)
O10—C4—C5—C6	−52.9 (4)	C8—C1—O9—C5	63.7 (3)
C3—C4—C5—C6	68.3 (4)	C2—C1—O9—C5	−62.8 (3)
O9—C5—C6—C7	52.5 (4)	C4—C5—O9—C1	67.1 (3)
C4—C5—C6—C7	−72.9 (4)	C6—C5—O9—C1	−63.2 (4)

### (1R*,2R*,5R*,6S*)-6-Bromo-9-oxabicyclo[3.3.1]nonan-2-ol Hydrogen-bond geometry (Å, º)

**Table:**

*D*—H···*A*	*D*—H	H···*A*	*D*···*A*	*D*—H···*A*
C2—H2*B*···O10^i^	0.95 (4)	2.45 (4)	3.378 (5)	164 (3)
O10—H10···O9^ii^	0.83 (6)	1.91 (6)	2.732 (4)	166 (5)

Symmetry codes: (i) *x*, −*y*+1/2, *z*−1/2; (ii) −*x*+1, *y*−1/2, −*z*+1/2.

## References

[bb1] Detert, H., Antony–Mayer, C. & Meier, H. (1992). *Angew. Chem.* **104**, 755–757.

[bb2] Detert, H. & Meier, H. (1997*a*). *Liebigs Ann. Recl* pp. 1557–1563.

[bb3] Detert, H. & Meier, H., (1997*b*). *Liebigs Ann. Recl*, pp. 1565—1570.

[bb4] Detert, H., Rose, B., Mayer, W. & Meier, H. (1994). *Chem. Ber.* **127**, 1529–1532.

[bb5] Krämer, G., Detert, H. & Meier, H. (2009). *Tetrahedron Lett.* **50**, 4810–4812.

[bb6] Meier, H., Krämer, G. & Detert, H. (2009). *Heterocycles* **78**, 2201–2208.

[bb7] Schollmeyer, D., Heidrich, M. & Detert, H. (2020). *IUCrData* **5**, x201302. 10.1107/S2414314620013024 PMC9462276 36338907

[bb8] Sheldrick, G. M. (2015*a*). *Acta Cryst.* A**71**, 3–8.

[bb9] Sheldrick, G. M. (2015*b*). *Acta Cryst.* C**71**, 3–8.

[bb10] Spek, A. L. (2009). *Acta Cryst.* D**65**, 148–155. 10.1107/S090744490804362X PMC2631630 19171970

[bb11] Stoe & Cie (2020). *X-RED* and *X-AREA*. Stoe & Cie, Darmstadt, Germany.

[bb12] Takahashi, A., Aso, M., Tanaka, M. & Suemune, H. (2000). *Tetrahedron* **56**, 1999–2006.

[bb13] Yates, P., Lewars, E. G. & McCabe, P. H. (1972). *Can. J. Chem.* **50**, 1548–1556.

